# The four-celled Volvocales green alga *Tetrabaena socialis* exhibits weak photobehavior and high-photoprotection ability

**DOI:** 10.1371/journal.pone.0259138

**Published:** 2021-10-26

**Authors:** Asuka Tanno, Ryutaro Tokutsu, Yoko Arakaki, Noriko Ueki, Jun Minagawa, Kenjiro Yoshimura, Toru Hisabori, Hisayoshi Nozaki, Ken-ichi Wakabayashi

**Affiliations:** 1 Laboratory for Chemistry and Life Science, Institute of Innovative Research, Tokyo Institute of Technology, Yokohama, Japan; 2 School of Life Science and Technology, Tokyo Institute of Technology, Yokohama, Japan; 3 Division of Environmental Photobiology, National Institute for Basic Biology, Okazaki, Japan; 4 Faculty of Life Science, Department of Basic Biology, The Graduate University for Advanced Studies, SOKENDAI, Okazaki, Japan; 5 Department of Biological Sciences, Graduate School of Science, The University of Tokyo, Tokyo, Japan; 6 Science Research Center, Hosei University, Tokyo, Japan; 7 Department of Machinery and Control Systems, College of Systems Engineering and Science, Shibaura Institute of Technology, Saitama, Japan; 8 Biodiversity Division, National Institute for Environmental Studies, Tsukuba, Japan; University of Innsbruck, AUSTRIA

## Abstract

Photo-induced behavioral responses (photobehaviors) are crucial to the survival of motile phototrophic organisms in changing light conditions. Volvocine green algae are excellent model organisms for studying the regulatory mechanisms of photobehavior. We recently reported that unicellular *Chlamydomonas reinhardtii* and multicellular *Volvox rousseletii* exhibit similar photobehaviors, such as phototactic and photoshock responses, via different ciliary regulations. To clarify how the regulatory systems have changed during the evolution of multicellularity, we investigated the photobehaviors of four-celled *Tetrabaena socialis*. Surprisingly, unlike *C*. *reinhardtii* and *V*. *rousseletii*, *T*. *socialis* did not exhibit immediate photobehaviors after light illumination. Electrophysiological analysis revealed that the *T*. *socialis* eyespot does not function as a photoreceptor. Instead, *T*. *socialis* exhibited slow accumulation toward the light source in a photosynthesis-dependent manner. Our assessment of photosynthetic activities showed that *T*. *socialis* chloroplasts possess higher photoprotection abilities against strong light than *C*. *reinhardtii*. These data suggest that *C*. *reinhardtii* and *T*. *socialis* employ different strategies to avoid high-light stress (moving away rapidly and gaining photoprotection, respectively) despite their close phylogenetic relationship.

## Introduction

Light is the essential energy source for phototrophic organisms. However, excess light energy leads to the production of toxic reactive oxygen species and possibly to cell death [1]. Phototrophic organisms have several photoprotection systems to prevent this negative aspect of light, which are induced by light as a signal. Non-photochemical quenching (NPQ) is such a system in the chloroplasts of plants and algae, which mitigates photo-induced oxidative stress [2]. The major component of NPQ is the energy-dependent quenching (qE) that dissipates excess light energy absorbed by photosystems through conversion of light as heat. In the model unicellular green alga *Chlamydomonas reinhardtii*, qE is facilitated by LHCSR (light harvesting complex, stress related) proteins, whose expression is triggered by light illumination [3,4] or exposure to low temperature [5]. In contrast, constitutively expressed PsbS protein regulates qE in the vascular plants and thus qE is rapidly induced [6].

Unlike immotile land plants, motile phototrophic organisms can physically move in response to light [7] and locate themselves under suitable light conditions for photosynthesis. Such behaviors are called photo-induced behavioral responses or photobehaviors. Typical photobehaviors include phototaxis and the photoshock response. Phototaxis describes the behavior of an organism moving toward or away from a light source (called positive and negative phototaxis, respectively). When an organism stops moving or reverses the motion direction upon a sudden change in light intensity, the behavior is referred to as the photoshock response. Both behaviors are widely observed among algae and are thought to be critical for survival.

**Fig 1:**
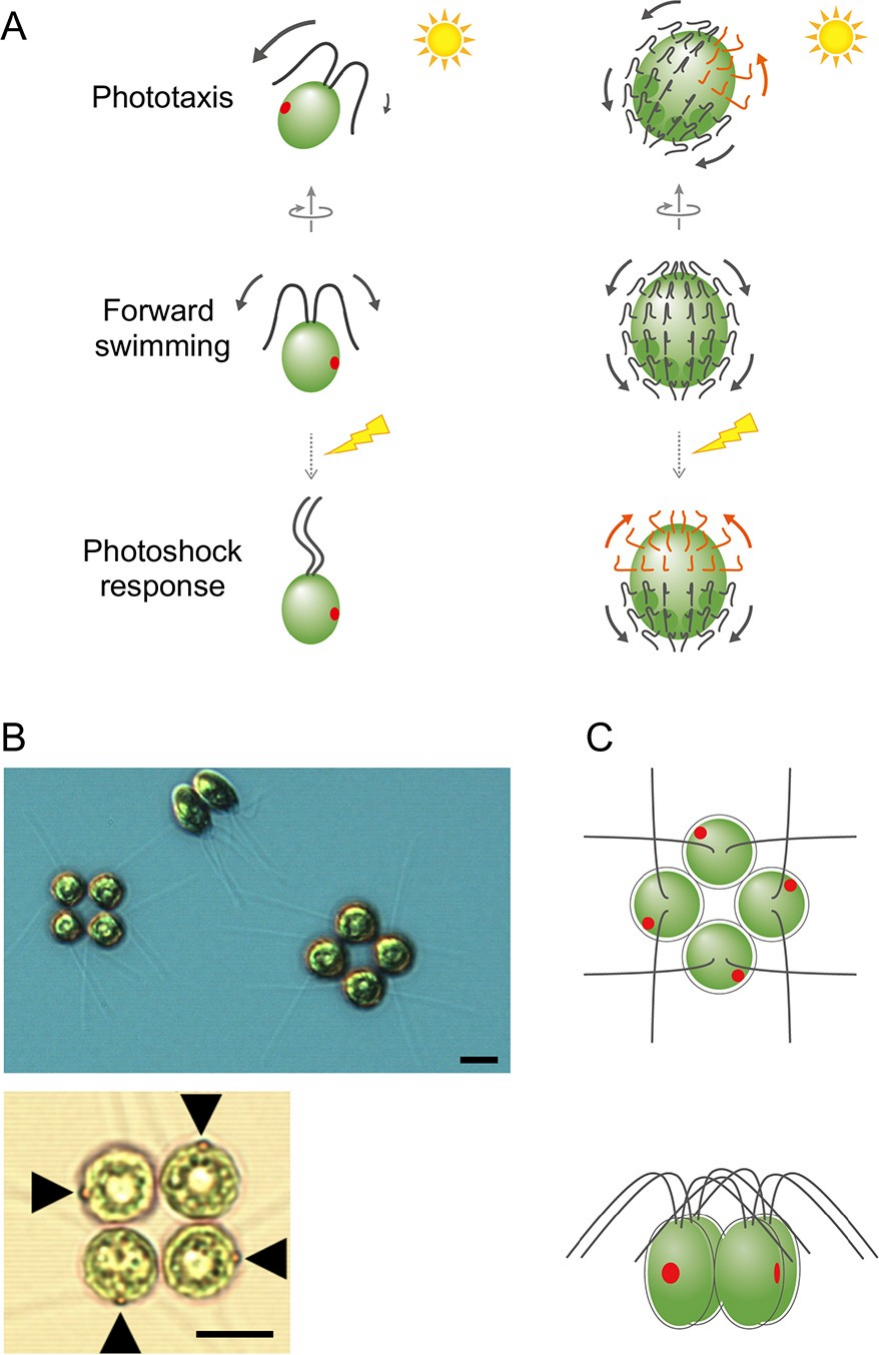
*Tetrabaena socialis* images and schematics of photobehavior in related volvocine species. (A) Schematics of photobehaviors in *C*. *reinhardtii* (left) and *Volvox rousseletii* (right). In *C*. *reinhardtii*, two cilia beat in opposite directions to propel the cell forward. For phototaxis, the force generated by two cilia becomes unbalanced, allowing the cell to change its swimming direction. For the photoshock response, the cilia waveform changes from asymmetrical to symmetrical, allowing the cell to swim backward. In *V*. *rousseletii*, all cilia beat toward the posterior pole of the spheroid for forward swimming. For phototaxis, the beating direction of cilia in the anterior hemisphere on the light-source side reverses. The force generated by cilia between the light-source hemisphere and the opposite hemisphere becomes imbalanced, changing the swimming direction of the spheroid. For the photoshock response, the beating direction of almost all cilia in the anterior hemisphere reverses. The forces generated by the anterior hemisphere and posterior hemisphere are balanced, stopping the spheroid motion. (B) A differential-interference-contrast (top) and a bright-field (bottom) images and (C) schematics of *T*. *socialis* NIES-571 colonies. Four cells are arranged in a square. Each *T*. *socialis* cell resembles a *C*. *reinhardtii cell*, possessing two cilia and one eyespot (arrowheads in the bottom panel). Scale bars: 10 μm.

Volvocine green algae are excellent model organisms for investigating photobehavior mechanisms. Among them, unicellular *Chlamydomonas reinhardtii* has long served as a reference organism for studying photobehavior, photosynthesis, and cilia (a.k.a. flagella) [8–11]. Each *C*. *reinhardtii* cell possesses one eyespot for photoreception and two cilia that generate force for swimming. The molecular mechanisms of *C*. *reinhardtii* photobehavior have been described in detail. Typically, the cilia beat ahead of the cell in opposite directions, allowing the cell to move forward using a movement pattern akin to the human breaststroke (Fig 1A, left). When the eyespot senses light, cation influx via channelrhodopsins (ChRs) localized at the eyespot trigger intraciliary Ca^2+^ concentration increases [12]. The cilia then change their beating mode in a Ca^2+^-dependent manner, and the cell exhibits the photobehavior. Phototaxis occurs when cells sense relatively weak light stimulation, raising the intraciliary Ca^2+^ concentration to ~10^−7^ M [13]. The beating frequency and amplitude of the cilia become unbalanced, and the difference in force generated by each cilium results in a directional change in the cell’s motion [14,15]. The photoshock response occurs when cells sense strong light stimulation, which induces depolarization of the plasma membrane and raises the intraciliary Ca^2+^ concentration to ~10^−4^ M [16]. The waveform of cilia then converts from asymmetrical to symmetrical, allowing the cell to swim backward with the cell body ahead of the cilia [17,18].

Like *C*. *reinhardtii*, multicellular volvocine algae are excellent organisms for studying photobehavior mechanisms. *Volvox* species are colonial algae, with thousands of cells comprising a single large spheroid. Each *Volvox* cell looks similar to *C*. *reinhardtii*, possessing one eyespot and two cilia. However, unlike *C*. *reinhardtii*, the two cilia on each cell beat in the same direction, i.e., to the spheroid’s posterior pole (Fig 1A, right) [19]. Upon photoreception at the eyespot, cilia of the cells stop beating (e.g., *Volvox carteri*) or rotate their beating plane without changing the waveform [e.g., *V*. *rousseletii*) to change the spheroid direction of motion [20–23]. Within *Volvox* spheroids, cells are gradually differentiated from the anterior pole to the posterior pole. In the case of *V*. *rousseletii*, the rotation angle of the beating plane is ~180° at the anterior pole, ~90° degrees at the equator, and ~0° degrees at the posterior pole [20,23]. Because of this differentiation, the anterior hemisphere is specialized for steering, while the posterior hemisphere is specialized for force generation. This steering and force labor differentiation enables *Volvox* spheroids to rapidly exhibit photobehaviors.

Volvocine algae evolution is unique in that modern multicellular (i.e., *Volvox*) and unicellular (i.e., *Chlamydomonas*) taxa both evolved from a *Chlamydomonas*-like unicellular ancestor, showing morphological diversity within the clade [24–26]. Additionally, *C*. *reinhardtii* and *Volvox* spp. exhibit vastly different regulatory mechanisms for photobehaviors, as described above. Thus, raising the question of how these differences arose during the evolution of multicellularity.

*Tetrabaena socialis* is a volvocine alga, described as the simplest known multicellular organism, comprised of only four cells [27,28]. *T*. *socialis* looks like four *C*. *reinhardtii* cells arranged in the same orientation, forming a square (Fig 1B and 1C). In this study, we analyzed *T*. *socialis* photobehaviors to elucidate the first step in the evolution of photobehavior regulation in multicellular volvocine algae. Surprisingly, unlike *C*. *reinhardtii* or *Volvox* spp., *T*. *socialis* does not exhibit clear phototaxis or photoshock responses. Instead, we observed a slow accumulation of *T*. *socialis* colonies toward the light source in a photosynthesis-dependent manner. Electrophysiological analyses revealed that the *T*. *socialis* eyespot does not function as a photoreceptor. Intriguingly, NPQ (qE) of *T*. *socialis* is constitutive like plants and higher than that observed in *C*. *reinhardtii*. These data suggest that *T*. *socialis* has a more effective photoprotective system than *C*. *reinhardtii*. Although *T*. *socialis* and *C*. *reinhardtii* belong to the same order, our findings suggest they employ diverse survival strategies.

## Results

### *T*. *socialis* does not exhibit significant photobehavior

Before comparing the photobehaviors of *T*. *socialis* and *C*. *reinhardtii*, we measured the swimming velocity and the ciliary beating frequency (CBF) of *T*. *socialis*, which were 137 ± 27 μm/sec and 16 ± 3 Hz, respectively (S1 Fig). Wild-type *C*. *reinhardtii* cells typically swim at ~150 μm/sec with a CBF of ~60 Hz [8]. If a sphere with a cross-sectional area about four times that of *C*. *reinhardtii* swims with the propulsive force of four *C*. *reinhardtii* cells, the estimated swimming velocity would be ~255 μm/sec, according to Stokes’ formula (S2 Fig). Thus, the actual swimming speed of *T*. *socialis* was slower than that predicted from its cell number and shape.

**Fig 2:**
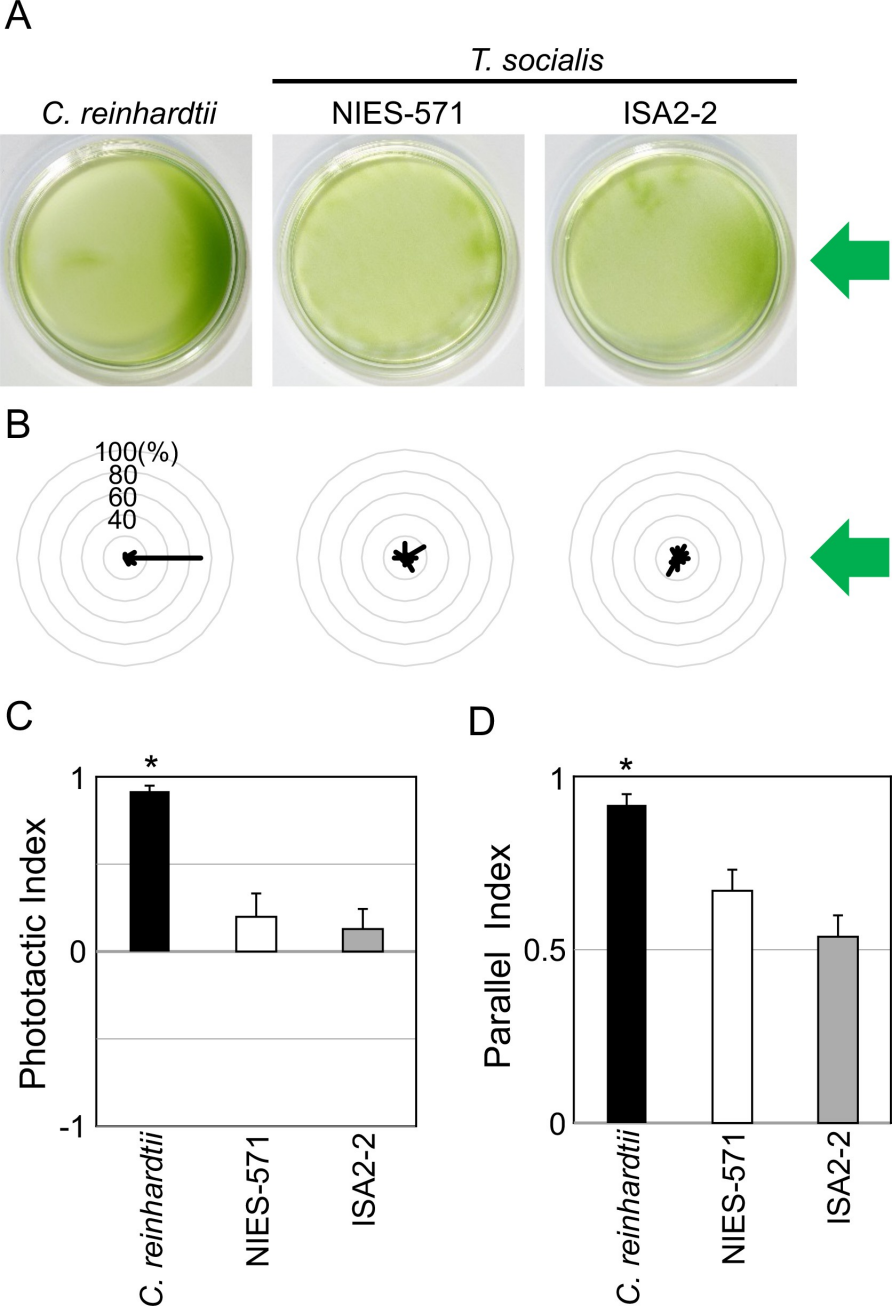
*Tetrabaena socialis* did not show phototaxis against a green light. (A) Suspensions of wild-type *C*. *reinhardtii* cells and two *T*. *socialis* strains, NIES-571 and ISA2-2, were illuminated with green light (λ = 525 nm, 30 μmol photons m^−2^ s^−1^) from the right side for 15 min. (B) Polar histograms representing the percentage of cells/colonies moving in a particular direction relative to the green light illumination from the right (12 bins of 30°; n = 30 cells/colonies per strain) for 3 s following a 30-s illumination. (C) Phototactic indices (average of cosθ) calculated from (B). If all cells/colonies show positive or negative phototaxis, the value will be 1 or −1, respectively. If all cells/colonies swim in random directions, the value will be 0. Asterisk represents a significant difference from random swimming (*p* < 0.01; Student’s *t*-test). (D) Parallel indices (average of |cosθ|) calculated from (B). If all cells/colonies exhibit phototaxis (positive or negative) or swim in a completely random direction, the values would be 1 or 0.622, respectively. Asterisk represents a significant difference from random swimming (*p* < 0.01; Student’s *t*-test).

To investigate *T*. *socialis* photobehaviors, we tested phototaxis using two strains of *T*. *socialis* (NIES-571 and ISA2-2) and wild-type *C*. *reinhardtii*. Algae cultures were placed in Petri dishes and illuminated with green LED (λ = 525 nm, 30 μmol photons m^−2^ s^−1^), which prominently induces phototaxis in *C*. *reinhardtii* (Fig 2A) [29–31]. However, no significant accumulation toward the light source was observed in *T*. *socialis* NIES-571, and only a slight accumulation was observed in ISA-2-2 (Fig 2A). We also observed the motion of *T*. *socialis* colonies (S1 Movie) and *C*. *reinhardtii* cells (S2 Movie) under a microscope and measured the angle between the light beam and the swimming trajectories. Still, we did not observe phototactic motion in *T*. *socialis* colonies in contrast to *C*. *reinhardtii* cells (Fig 2B). Phototactic indices (to examine whether colonies showed positive or negative phototaxis) and parallel indices (to examine whether colonies swam in parallel to the light beam) calculated from those angles provided no evidence of phototaxis in *T*. *socialis* colonies (Fig 2C and 2D). Thus, the slight photoaccumulation (accumulation of organisms in the presence of light) observed in ISA2-2 may be attributed to a phenomenon other than phototaxis.

**Fig 3:**
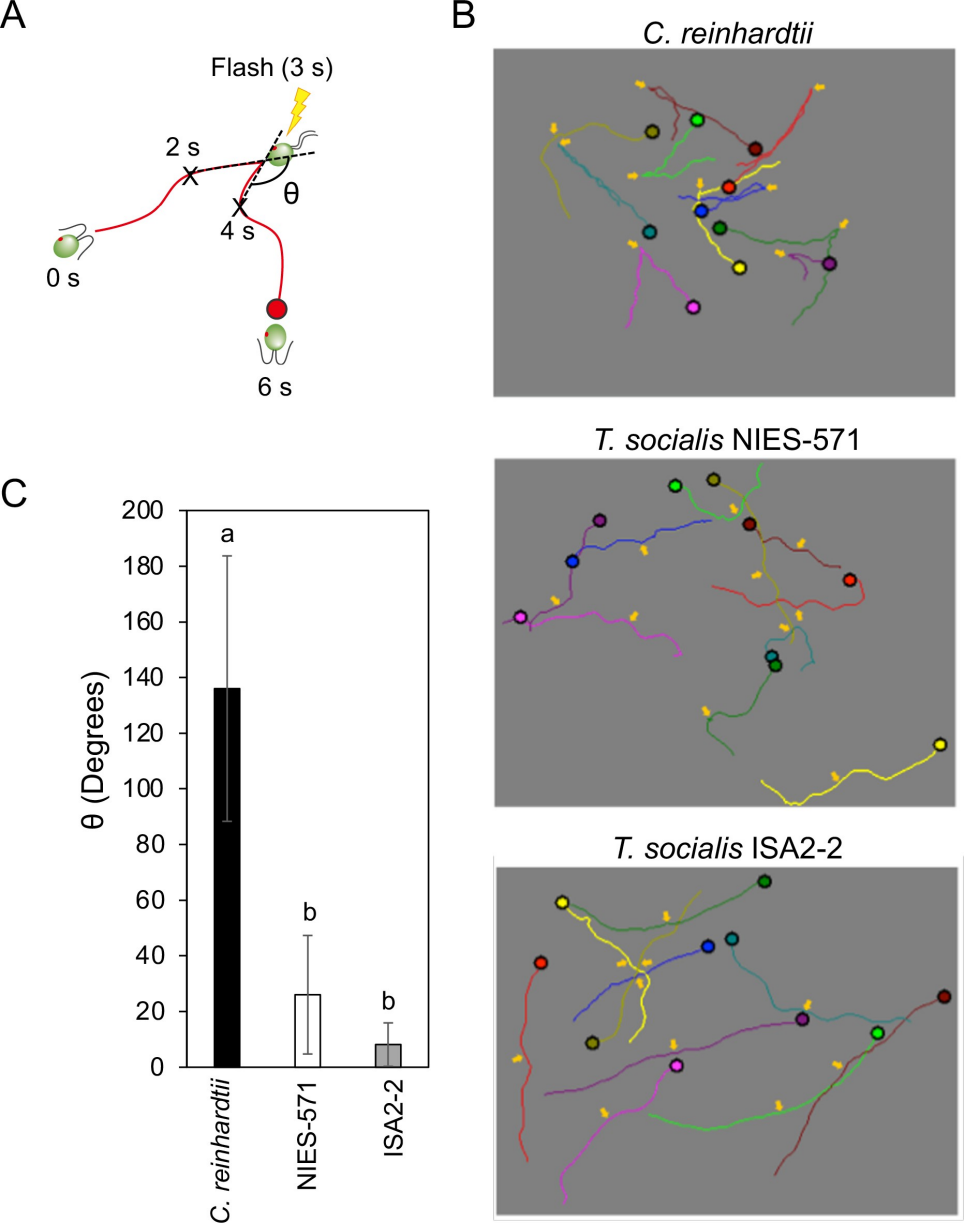
*Tetrabaena socialis* did not exhibit a photoshock response. (A) Schematic image of the photoshock response in *C*. *reinhardtii*. After a flash illumination, *C*. *reinhardtii* cells change their ciliary waveform, swimming backward for a short period before returning to their normal swimming pattern. Trajectories for 6 s were traced before and after a flash illumination, and the angle (θ) between trajectories before and after the flash illumination for 1-s each was measured. (B) Representative 6-s swimming trajectories of wild-type *C*. *reinhardtii* cells and *T*. *socialis* NIES-571 and ISA2-2 colonies. Different colors indicate different cell trajectories. The yellow arrows indicate the flashlight illumination point. (C) The angles between trajectories before and after the flash illumination. Means ± S.D. (n = 10 cells/colonies) are shown. Different letters indicate significant differences (*p* < 0.01, one-way ANOVA and Tukey honest significance difference [HSD]).

Next, we tested the photoshock responses of *T*. *socialis* and *C*. *reinhardtii*. Changes in the angles (θ) between swimming trajectories of cells/colonies before and after the flash illumination were measured (Fig 3A). Immediately after the flash illumination, *C*. *reinhardtii* cells swam backward for a short period (θ = 136.0 ± 47.7 degrees) (Fig 3B and 3C and S3 Movie) [26]. The swimming trajectories of *C*. *reinhardtii* cells formed V-shapes with the apex at the flash illumination point (Fig 3B). In contrast, *T*. *socialis* colonies did not change the swimming direction as *C*. *reinhardtii* upon the flash illumination (θ = 26.0 ± 6.7 degrees for NIES-571; 8.2 ± 2.5 degrees for ISA2-2) (Fig 3B and 3C and S4 Movie). These data provided no evidence of the photoshock response in *T*. *socialis* colonies.

Summarizing the results so far, *T*. *socialis* colonies do not exhibit immediate photobehaviors after light stimulation in contrast with volvocine relatives such as *C*. *reinhardtii* (unicellular), *Gonium pectrale* (16 cells), *Volvox carteri* (~2,000 cells), and *Volvox rousseletii* (~5,000 cells) [20,22,32–35].

### Photoreceptor currents not detected in *T*. *socialis* eyespots

*C*. *reinhardtii* photobehaviors occur via changes in the cilia beating patterns after photo-sensing at the eyespot [32,35]. Thus, we proposed that the lack of photobehavior in *T*. *socialis* could be due to defects in the cilia or the eyespot.

To test whether *T*. *socialis* cilia possess the waveform conversion ability that enables the *C*. *reinhardtii* photobehaviors, we demembranated *T*. *socialis* colonies using non-ionic detergent. After preparing the demembranated cell models, we reactivated their motility by adding ATP in a high-Ca^2+^ buffer [13,23]. The cilia of live *T*. *socialis* showed asymmetrical waveform, whereas demembranated *T*. *socialis* cilia showed symmetrical waveform in the 10^−3^ mM Ca^2+^ buffer (S3 Fig). These data show that *T*. *socialis* cilia have retained the Ca^2+^-dependent waveform conversion capability. Observations of spontaneous backward swimming in live *T*. *socialis* colonies further support this finding (S5 Movie).

**Fig 4:**
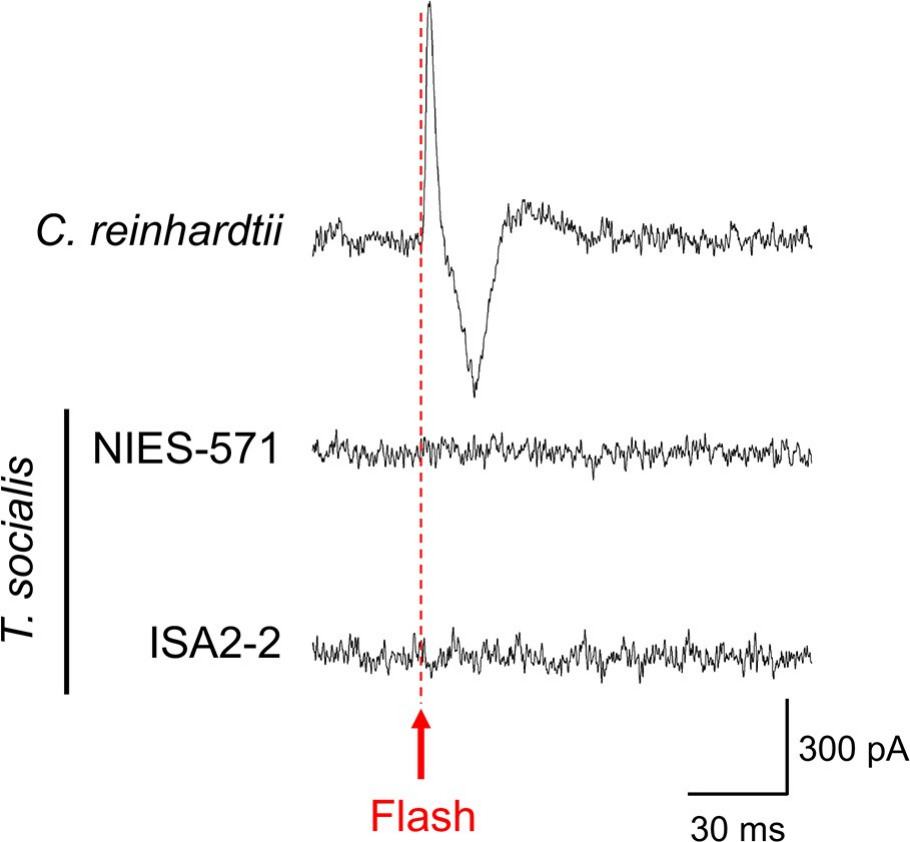
Photoreceptor currents were not detected in *Tetrabaena socialis* after a flash illumination. Representative photoreceptive currents in wild-type *C*. *reinhardtii* cells (1.0 × 10^7^ cells/mL) and NIES-571 and ISA2-2 *T*. *socialis* colonies (2.5 × 10^6^ colonies/mL). The flashlight illumination is indicated with a red arrow and a broken line. The experimental setup is shown in S4 Fig.

Next, we tested whether defects in the eyespot were responsible for the lack of photobehaviors in *T*. *socialis*. The volvocine algae eyespot comprises two parts: ChRs functioning as photoreceptors and carotenoid-rich granule layers acting as light reflectors [30,36,37]. In *C*. *reinhardtii*, the light-activated cation channel activities of ChRs can be electrophysiologically detected as photoreceptor currents (PRC) (S4 Fig) [38]. Each *T*. *socialis* cell contains one detectable red spot, and a previous study confirmed that the eyespot structure is intact (Fig 1B) [27]. We measured the PRC of two strains of *T*. *socialis* and *C*. *reinhardtii* as a control. *C*. *reinhardtii* cells showed a prominent PRC signal after a flash illumination, which is similar to the previous studies using the same experimental setup (Fig 4) [38,39]. However, no PRC signal was detected in either *T*. *socialis* strain (Fig 4). These data suggest that the *T*. *socialis* cell eyespots do not function as photoreceptors. A ChR ortholog was identified in the *T*. *socialis* genome data [40]; however, the amino-acid substitution in the retinal-binding domain could be responsible for the eyespot dysfunction (S5 Fig).

### Photosynthesis-dependent photoaccumulation of *T*. *socialis*

As described above, *T*. *socialis* did not display typical photobehaviors. However, the ISA2-2 strain colonies accumulated slowly toward the green light source (photoaccumulation) without showing phototaxis (Fig 2A). The absorption peak of *C*. *reinhardtii* ChR1 is ~470 nm, which is close to a chlorophyll b absorption peak (~460 nm) [12,41]. As photosynthetic activity modulates phototaxis signals, green light (~525 nm) is preferred for phototaxis analyses to minimize the effects of photosynthesis [42,43].

**Fig 5:**
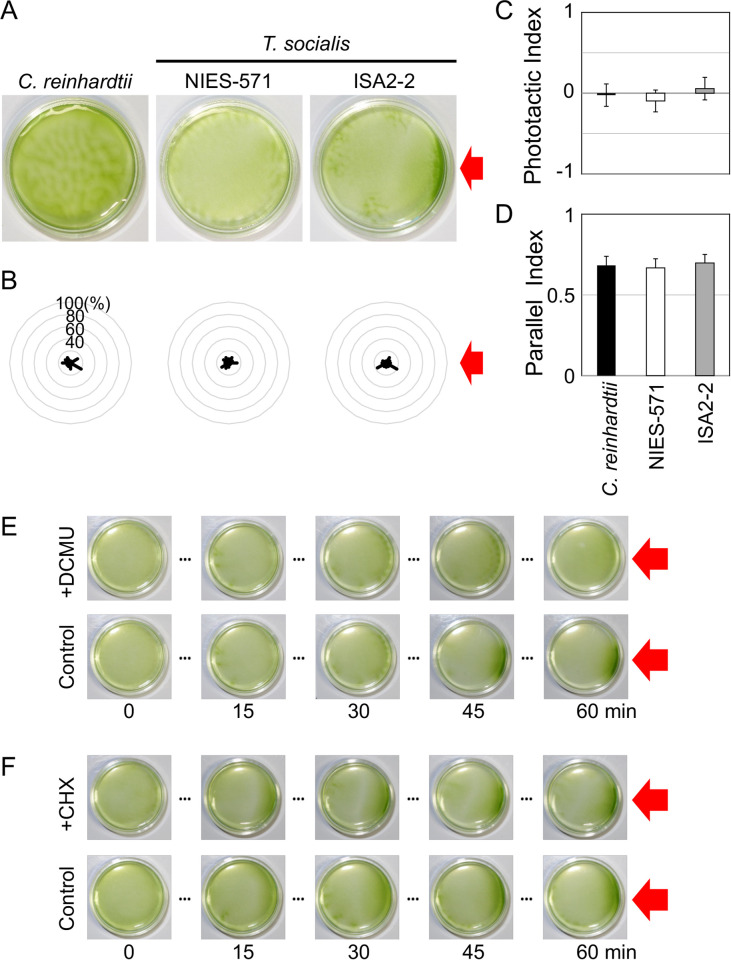
*Tetrabaena socialis* ISA-2-2 showed slow photoaccumulation toward red light in a photosynthesis-dependent manner. (A) Suspensions of wild-type *C*. *reinhardtii* and *T*. *socialis* NIES-571 and ISA2-2 strains were illuminated with red light (λ = 640 nm, 30 μmol photons m^−2^ s^−1^) from the right side for 15 min. (B) Polar histograms representing the percentage of cells/colonies moving in a particular direction relative to the red light illumination (12 bins of 30°; n = 30 cells/colonies per strain) for 3 s following 30-s illumination. (C) Phototactic indices (average of cosθ) and (D) parallel indices (average of |cosθ|) calculated from (B). Values were not significantly different from random swimming (*p* > 0.1; Student’s *t*-test; see Fig 2C and 2D for details). (E, F) Time-lapse observation of *T*. *socialis* ISA2-2 photoaccumulation with (E) DCMU (or ethanol as a control) or (F) cycloheximide (CHX) (or DMSO as a control) treatments for 15 min in the dark. After the red light illumination, images of the Petri dishes were captured every 15 min.

We surmised that ISA2-2 photoaccumulation may be related to photosynthesis and conducted a phototaxis assay using a red LED (λ = 640 nm, 30 μmol photons m^−2^ s^−1^) to test our hypothesis. When we used red light for illumination, ISA2-2 colonies accumulated toward the light source in ~15 min but *C*. *reinhardtii* cells and NIES-571 colonies did not (Fig 5A). Our cell/colony-level observations suggest that the accumulation could not be attributed to phototaxis: neither the phototactic index nor the parallel index in ISA2-2 showed significant difference from the random swimming (Fig 5B–5D). Interestingly, the photoaccumulation of ISA2-2 was inhibited by treatment with photosynthesis inhibitor DCMU (Fig 5E) but not ribosome inhibitor cycloheximide (CHX) (Fig 5F). Together, these findings indicate that photoaccumulation of *T*. *socialis* ISA2-2 toward red light is photosynthesis-dependent and does not require *de novo* protein expression.

So, how do *T*. *socialis* ISA2-2 colonies photoaccumulate without showing phototaxis or photoshock responses? We hypothesized that colonies might alter their swimming trajectory angle depending on the light intensity, as observed in the photoaccumulation of *Euglena gracilis* [44]. If the angle does not change when a colony moves from the dark to the light area and changes when a colony moves out from the light to the dark area, the number of colonies in the light area may increase over time (S6A Fig). However, cell trajectory analyses at the border of the light and dark areas provided no evidence for this hypothesis. (S6B Fig). We could not clarify the mechanism of photosynthesis-dependent photoaccumulation in *T*. *socialis*.

### High non-photochemical quenching (qE) in *T*. *socialis*

Photobehaviors are believed to be crucial for survival under changing light environments. In particular, strong light conditions can lead to photo-oxidative damage in the phototrophic organisms, resulting in excessive reactive oxygen species generation [1]. We predicted that *T*. *socialis* might have a more effective photoprotection system than volvocine relatives, allowing the species to survive in environments with strong and varied light without showing immediate photobehaviors.

**Fig 6:**
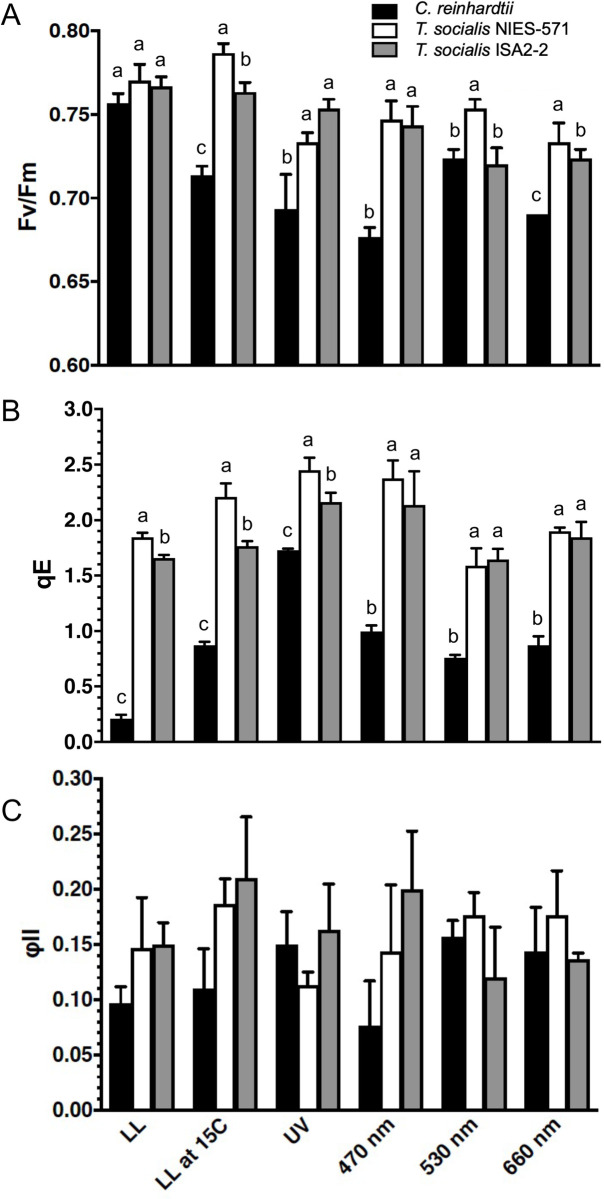
Photosynthetic parameters of *C*. *reinhardtii* and *T*. *socialis under various light conditions*. Fv/Fm (A), NPQ (qE) (B), and φII (C) of wild-type *C*. *reinhardtii* and *T*. *socialis* NIES-571 and ISA2-2 strains were measured by a pulse-amplitude modulation chlorophyll fluorometer. Cells/colonies were illuminated under various light conditions (LL: white fluorescent light at 20 μmol photons m^−2^ s^−1^; UV fluorescent light at 20 μmol photons m^−2^ s^−1^; 470-, 530-, 660-nm LED light at 200 μmol photons m^−2^ s^−1^) for 4 h prior to the analysis. Means ± S.D. of three independent experiments are shown. In (A) and (B), different letters indicate significant differences (*P* < 0.01, one-way ANOVA and Tukey honest significance difference [HSD]). In (C), no significant difference between any two groups was found in each parameter (*P* > 0.01, one-way ANOVA and Tukey honest significance difference [HSD]).

NPQ is a typical chloroplast photoprotection system that dissipates excess absorbed energy from the photosystems [2,45]. The major component of NPQ is energy-dependent qE [46], facilitated by the LHCSR protein expression in green algae [47,48]. In *C*. *reinhardtii*, the expression of LHCSR3 and LHCSR1, the major factors for qE, is induced by blue or UV illumination, respectively [3,48]. To evaluate the qE capability of the algae as well as the photosynthetic capability, we illuminated cells with UV, blue, green, or red light. The low-temperature exposure was also tested to evaluate the effects of growth temperature difference in *C*. *reinhardtii* (23°C) and *T*. *socialis* (15°C). Following these treatments, we measured qE as well as other photosynthetic parameters Fv/Fm (maximal photochemical efficiency of PSII representing photosynthetic efficiency) and φII (effective quantum yield of PSII) by a chlorophyll fluorometer (Fig 6). Under visible high-light conditions, Fv/Fm values in *T*. *socialis* were significantly higher than in *C*. *reinhardtii*, especially at 470 nm and 660 nm (Fig 6A). Moreover, qE values in *T*. *socialis* were relatively stable among the conditions and significantly higher than those of *C*. *reinhardtii* under all light and temperature conditions (Fig 6B). In contrast, φII values were not different between species under all conditions tested (Fig 6C), suggesting that the higher Fv/Fm values in *T*. *socialis* than *C*. *reinhardtii* were not due to the differences in photochemical quenching. Altogether, these data suggest that *T*. *socialis* possesses a higher photoprotection ability than *C*. *reinhardtii*.

**Fig 7:**
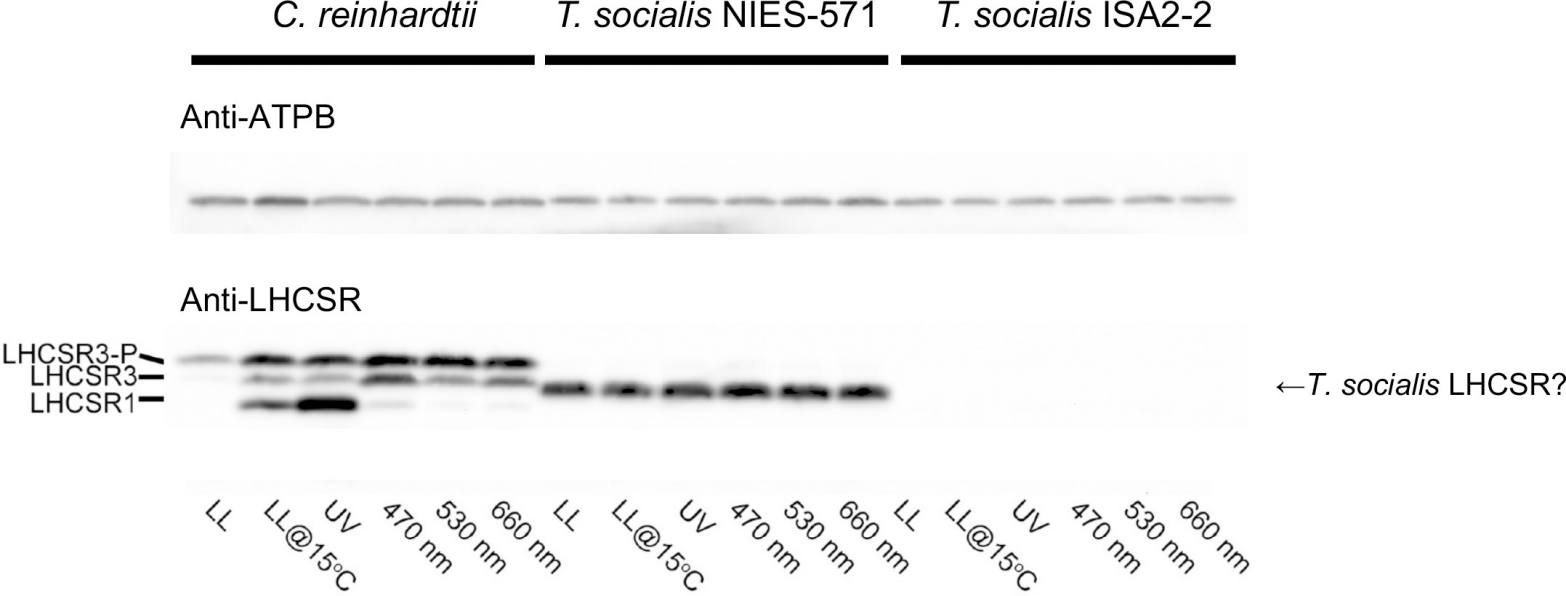
Expression of LHCSR proteins *in C*. *reinhardtii* and *T*. *socialis under various light conditions*. Western blotting against whole-cell extracts (WCE) of wild-type *C*. *reinhardtii* and *T*. *socialis* NIES-571 and ISA2-2 strains using the antibody to detect *C*. *reinhardtii* LHCSR proteins (lower panel) or anti-ATPB (the beta subunit of ATP synthase) (upper panel) as a loading control. WCE were prepared after the same light/temperature treatment as Fig 6. LHCSR3-P: phosphorylated LHCSR3.

These qE phenomena imply that *T*. *socialis* might constitutively accumulate LHCSR proteins, where *C*. *reinhardtii* does not. To test this idea, we assessed the expression level of LHCSR proteins in the algae under the same light/temperature conditions as used in Fig 6 by western blotting (Fig 7). LHCSR3- and LHCSR1-orthologs are present in *T*. *socialis* transcriptome database constructed by the data from NIES-571 [40], though the LHCSR3-like protein in *T*. *socialis* is slightly smaller than *C*. *reinhardtii* LHCSR3 (S7 Fig). These proteins possess a peptide sequence similar to the epitope of the antibody that detects both *C*. *reinhardtii* LHCSR3 and LHCSR1 (S7 Fig) [49]. As reported previously, the expression of LHCSR3 and LHCSR1 in *C*. *reinhardtii* was prominently induced by blue or UV illumination, respectively [3,48], and slightly by low light illumination at 15°C (Fig 7) [5]. In contrast, in *T*. *socialis* NIES-571, the band reacted with the anti-LHCSR protein antibody (probably two bands of LHCSR3-like and LHCSR-1 like proteins overlap) were detected in all the conditions tested, even without blue- or UV-light illumination (Fig 7). Moreover, in *T*. *socialis* ISA2-2, the bands were not detected in all the conditions (Fig 7). Together with the qE data in Fig 6B, these data suggest that molecular mechanisms to facilitate qE are different between *C*. *reinhardtii* and *T*. *socialis* and even between the strains in *T*. *socialis*.

## Discussion

Our findings revealed that *T*. *socialis* does not display immediate photobehaviors after light illumination like *C*. *reinhardtii* and *Volvox* species. However, the photoprotection ability of *T*. *socialis*, represented by NPQ (qE), is higher than that of *C*. *reinhardtii*. These data indicate that *T*. *socialis* and *C*. *reinhardtii* employ different survival strategies, despite being closely related.

### Weak photobehaviors of *T*. *socialis*

*T*. *socialis* did not show immediate photobehaviors, which occur within ~1 s after photostimulation in *C*. *reinhardtii* and *Volvox* species (Figs 2 and 3) [20,35,50]. In *C*. *reinhardtii* and *Volvox*, the photobehavior mechanism involves Ca^2+^-dependent cilia movement pattern alterations after photostimulation [15,17,23,32,51]. The results of our demembranated model experiment suggest that *T*. *socialis* cilia have retained the ability for Ca^2+^-dependent waveform conversion (S3 Fig). Thus, the lack of immediate photobehaviors in *T*. *socialis* might be attributed to the loss of PRC (Fig 4). Comparing the amino-acid sequences of *T*. *socialis*, *C*. *reinhardtii*, *V*. *carteri*, and *Gonium pectrale* (S5 Fig), we found an amino-acid substitution in the ChR1 gene [12,52]. The mutated amino acid contributes to retinal binding in ChR [53], which may cause ChR dysfunction in *T*. *socialis*.

Although *T*. *socialis* did not show an immediate photoresponse, the ISA2-2 strain exhibited slow photoaccumulation in a photosynthesis-dependent manner (Fig 5). However, no evidence of phototaxis or photoshock responses was observed, and we could not clarify the underlying mechanism of the observed photoaccumulation. Time-lapse colony observations in a wide field of view will be necessary to elucidate how the colonies respond to light and change their swimming directions. Among the two *T*. *socialis* strains tested, NIES-571 did not show slow photoaccumulation (Fig 5). This ability may have been lost during the domestication of long-term cultures in the laboratory [9], as NIES-571 was isolated in 1982, while ISA2-2 was isolated in 2014.

Some photosynthesis-dependent cellular motions have been previously reported, such as movements of the nucleus and mitochondria in *Arabidopsis thaliana* cells [54,55]; however, the direct signal regulating the motion is unclear. In this study, the photoaccumulation of ISA2-2 was not inhibited by cycloheximide, suggesting that it was not induced by “retrograde signaling,” wherein nucleus-encoded gene expression is regulated by signals from the chloroplast [56]. Reactive oxygen species have been reported to regulate ciliary beatings and phototactic signals [29,57], representing a candidate for further study on the photoaccumulation mechanism in ISA2-2.

### High and immediate NPQ induction in *T*. *socialis*

*T*. *socialis* exhibited higher photosynthetic efficiency (Fv/Fm) under high-light stress conditions and higher NPQ (qE) capability than *C*. *reinhardtii*, indicating a higher chloroplast photoprotection ability against strong light in *T*. *socialis* (Fig 6A and 6B). Moreover, in *T*. *socialis*, LHCSR proteins were almost always expressed (NIES-571) or, surprisingly, totally not expressed (ISA2-2) (Fig 7).

To interpret the results of LHCSR expression in *T*. *socialis*, we considered two possibilities. First, qE is completely independent from LHCSR proteins in both strains of *T*. *socialis*, and LHCSR proteins in NIES-571 serve a different function from qE facilitation. Second, LHCSR proteins contribute to qE facilitation in NIES-571 strain, but their expression is constitutive regardless of light conditions. In this case, it is possible that ISA2-2 is a mutant with defects in LHCSR expression and some alternative pathway that facilitates qE is enhanced. In any case, further analyses will necessary to elucidate the difference of molecular mechanism of the qE facilitation in *T*. *socialis* and *C*. *reinhardtii*.

It is interesting to consider the weak-photobehavioral and high-photoprotective phenotypes in *T*. *socialis* in relation to its habitat. *T*. *socialis* was isolated from shallow freshwater areas or pools in Antarctic ice [58,59]. In such an environment, algae may be unable to escape or avoid strong sunlight by changing the swimming manner. We propose two hypotheses for the evolution of high-photoprotection function in *T*. *socialis*. First, *T*. *socialis* may have gained its high and constitutive photoprotection ability after multicellularity. The four-celled arrangement of *T*. *socialis* is not ideal for controlling swimming direction; thus, only those acquired mutations for high-photoprotection ability survived. Second, a *Chlamydomonas*-like unicellular ancestor may have gained high-photoprotection ability. Only the ancestor with this adaptation was able to evolve multicellularity, producing multicellular offspring that all possess high-photoprotection abilities, regardless of their photobehavior capabilities. In either case, *T*. *socialis* no longer required immediate photoresponses, and the ChR mutation may have been fixed.

The *T*. *socialis* phenotype paradoxically emphasizes the importance of immediate photobehaviors for photoprotection in microalgae. Photobehavior has been reported in various microalgae, though its importance in nature has not been clearly demonstrated. The negative correlation between photobehavior and photoprotection abilities in *C*. *reinhardtii* and *T*. *socialis* suggests that the photobehavior is essential for protecting photosystems in environments where light conditions fluctuate. Unlike *C*. *reinhardtii*, NPQ (qE) in land plants can always be induced because PsbS, an essential NPQ component, is constitutive [6]. It is interesting to note that phototrophic organisms with low-motility properties (land plants) and with weak-photobehavior (*T*. *socialis*) share a similar photoprotection capability (constitutive NPQ (qE)). However, a recent study suggested that NPQ is constitutive even in *C*. *reinhardtii* under conditions that mimic the wild environment [60]. The negative correlation between *C*. *reinhardtii* and *T*. *socialis* in terms of photobehavior and photoprotection abilities, as well as contribution of photobehavior to survival in the wild environment, will need to be tested by further analyses.

In conclusion, our results showed that *T*. *socialis* does not show immediate photobehaviors after light illumination, which may be due to the loss of function of the eyespot. Instead, it has a high and constitutive photoprotection capability. These properties of *T*. *socialis* contrast with relatively lower and induced photoprotection capability in *C*. *reinhardtii*, which exhibits agile photobehaviors. This study suggests the diversity of survival strategies of phototrophic organisms that differ even among closely related species.

## Materials and methods

### Strains and culture

*T*. *socialis* NIES-571 and ISA2-2 were grown in standard Volvox medium (SVM) at 15°C under a 12 h/12 h light/dark cycle using 20 μmol photons m^−2^ s^−1^ white light [61]. NIES-571 was isolated from Kanagawa prefecture, Japan, in 1982 [62]. ISA2-2 was isolated from Saitama prefecture, Japan, in 2014. *C*. *reinhardtii* strain CC-125 was grown in tris-acetate-phosphate (TAP) medium at 25°C under a 12 h/12 h light/dark cycle using 20 μmol photons m^−2^ s^−1^ white light [63]. Before the photosynthetic parameter measurements, *C*. *reinhardtii* cells in the mid-log phase grown in TAP medium were collected a few hours after the beginning of the light period, resuspended in high-salt minimal medium (HSM) [64], and cultured by shaking at 23°C under a 12 h/12 h light/dark cycle for 24 h.

### Phototaxis assay and measurement of swimming velocity

The phototaxis assay was conducted following a previously described method with modifications [30]. In brief, *T*. *socialis* colonies and *C*. *reinhardtii* cells were washed with solution A (50 mM Hepes-NaOH pH 8.0, 2mM EGTA-NaOH pH 7.0, 10 mM KCl, 3 mM CaCl_2_) and solution B (50 mM Hepes-NaOH pH 7.4, 2mM EGTA-NaOH pH 7.0, 10 mM KCl, 3 mM CaCl_2_), respectively. For dish assays, colony/cell suspensions (1.0 × 10^7^ cells/ml for *C*. *reinhardtii* and 1.0 × 10^6^ colonies/ml for *T*. *socialis*) were added to Petri dishes (35-mm diameter, 10-mm depth), placed under dim red light for 15 min, illuminated with LEDs at ~30 μmol photons m^−2^ s^−1^ (green: λ = 525 nm; red: λ = 640 nm) from one side, and photographed (DSC-RX100M2; Sony). Cells/colonies were observed under a dark-field microscope (BX-53, Olympus) with dim red light (λ > 630 nm) and recorded using a CCD camera (1129HMN1/3; Wraymer) for single-colony/cell analyses. The angle (θ) between the light direction and the swimming direction was measured for 3 s, following side-illumination with LED for 45 s for *T*. *socialis* or 10 s for *C*. *reinhardtii*. Videos of swimming cells/colonies were auto-tracked using Image Hyper software (Science Eye), and angles were measured from the trajectories. Swimming velocity was calculated from the trajectories simultaneously. DCMU (3-(3,4-dichlorophenyl)-1,1-dimethylurea; #11828–82, Nacalai Tesque) was dissolved in ethanol, and cycloheximide (#06741–91, Nacalai Tesque) was dissolved in DMSO (#13445–74, Nacalai Tesque). The final reagent concentrations (0.1 mM DCMU and 10 μg/ml cycloheximide) were obtained from previous studies [42,65]. After adding DCMU or cycloheximide reagent, the colony/cell suspensions were placed in the dark for 15 min and then subjected to the phototaxis assay.

To observe the swimming trajectories of *T*. *socialis*, we observed the colony suspensions under a bright-field microscope (BX-53, Olympus) with white light (10 μmol photons m^-2^ s^-1^). Half of the view field was covered with an ND10 filter. Videos of the swimming colonies were auto-tracked using Image Hyper software (Science Eye) for 6 s (3 s before and 3 s after swimming across the light-dark border or 6 s in the same light area), and angles were measured from the trajectories.

### Photoshock response assay and measurement of ciliary beating frequency

*T*. *socialis* colonies and *C*. *reinhardtii* cells were washed using the method described for the phototaxis assay. The suspensions were kept under dim red light for 15 min, then cells/colonies were observed under a dark-field microscope (BX-53, Olympus) with dim red light (λ > 630 nm) and recorded using a high-speed camera (HAS-L2M, DITECT) at 400 fps. Videos of swimming cells/colonies were auto-tracked using Image Hyper software (Science Eye) and angles were measured from the trajectories before and after the flash illumination (white light; TT560 Speedlite, Neewer). Ciliary beating frequency was calculated from the time it takes for a cilium to beat once.

### Photocurrent measurements

PRCs were assessed in populations of *T*. *socialis* colonies and *C*. *reinhardtii* cells according to the method of Sineshchekov et al. (1992) with modifications [38,66]. In brief, 1 ml of the *T*. *socialis* colony suspension (2.5 × 10^6^ colonies/mL) or *C*. *reinhardtii* cell suspension (1.0 × 10^7^ cells/mL) in a measuring solution (0.5 mM Hepes, pH 8.0, 0.1 mM CaCl_2_) was added to a cuvette (10 × 10 × 15 mm) with one electrode on each side of its rectangular bottom. A flash light (λ = 500 nm, 1560 μmol photons m^−2^ s^−1^) was applied using an LED source (NSPE510S, Nichia Chemical) from one side of the electrode for 1 msec. The current was measured using a patch-clamp amplifier (Axoclamp 200B, Axon).

### Photosynthetic parameter measurements

One hour after the beginning of the light period, chlorophyll contents in each alga were measured by the method of [67]. *T*. *socialis colonies* in a late-log phase and *C*. *reinhardtii* cells after replacement to HSM (see “Strains and culture”) were harvested by centrifugation 720 × g for 3 min, resuspended in SVM (*T*. *socialis*) or HSM (*C*. *reinhardtii)* to the colony/cell density in that the chlorophyll concentration was 2.5 μgChl/mL and cultured again for 4 h for recovery from the centrifugation stress. Algal cells were then irradiated with low light (LL; white fluorescent light at 20 μmol photons m^−2^ s^−1^), UV light (low-level UV-supplemented fluorescent light provided by a ReptiSun10.0 UV fluorescent bulb [4] at 20 μmol photons m^−2^ s^−1^), or 470-, 530-, 660-nm LED lights at 200 μmol photons m^−2^ s^−1^ for 4 h. Total light intensities were measured using a sun spectroradiometer (S-2442 HIDAMARI mini, SOMA OPTICS, LTD.) with a 300–800 nm range. Chlorophyll fluorescence-based NPQ (qE) measurements were performed as follows. Maximum yields (Fm) were measured under dark conditions [after weak far-red (< 5 μmol m^−2^ s^−1^) treatment for 30 min] using Fluorocam (Photon System Instruments, Czech Republic). The maximum and steady-state fluorescence yields under light (Fm′ and F, respectively) were measured after actinic irradiation at 750 μmol m^−2^ s^−1^ for 30 s. Fv/Fm and φII were calculated using the equation Fv/Fm = (Fm − Fo)/Fm, φII = (Fm′ − F)/Fm′, respectively. NPQ (qE) was estimated using the equation NPQ  =  (Fm − Fm′)/Fm′.

### Western blotting of LHCSR proteins

Protein samples of whole-cell extracts (corresponding to ~2.0 × 10^6^ cells) were loaded onto 11% (w/v) acrylamide SDS-PAGE gels containing 7 M urea and blotted onto PVDF membranes. Antiserum against the beta subunit of ATP synthase (ATPB) control protein was obtained from Agrisera (AS05 085, rabbit polyclonal); antiserum against LHCSRs (detecting both LHCSR1 and LHCSR3) was raised and affinity-purified against the peptide LGLKPTDPEELK as reported previously [49]. An anti-rabbit horseradish peroxidase-conjugated antiserum (#7074, Cell Signaling Technology) was used as the secondary antibody. Blots were developed using EzWestLumi plus ECL detection reagent (ATTO), and images of the blots were obtained using a ChemiDocTouch System CCD imager (Bio-Rad Laboratories). The upper LHCSR3 band represents the phosphorylated form of LHCSR3 [3].

### Reactivation of demembranated cell models and ciliary waveform trace

For *C*. *reinhardtii*, cell models were prepared using a previously described method [13]. For *T*. *socialis*, colonies were harvested by centrifugation, washed with SVM without Ca(NO_3_)_2_ and HES (10 mM Hepes-NaOH pH7.4, 5 mM MgSO_4_, 1 mM EGTA-NaOH pH 7.0, 4% sucrose) sequentially, and suspended in a demembranation buffer (30 mM Hepes-NaOH pH 7.4, 5 mM MgSO_4_, 1 mM EGTA-NaOH pH 7.0, 50 mM K-acetate, 0.015% Igepal CA-630 (I3021; Sigma-Aldrich), and 0.25 M DTT). Demembranated cells/colonies were suspended in a reactivation buffer containing 10^−3^ M free Ca^2+^ [23] and reactivated with final 1 mM ATP. Most ciliary axonemes were detached, and freely swimming axonemes were observed under a dark-field microscope (BX-53, Olympus) and recorded using a high-speed camera (HAS-L2M, DITECT) at 400 fps. Live cells/colonies were trapped on a surface of a glass slide coated with 0.1% polyethylenimine, and the ciliary beatings were observed and recorded using the same observation setup as above. The video of ciliary/axonemal beatings were played back frame-by-frame and the waveforms were manually traced by the mouse using PowerPoint (Microsoft).

## Supporting information

S1 Fig Motility parameters of *T*. *socialis*. Ciliary beating frequency (left) and swimming velocity of *T*. *socialis* NIES-571 (n = 20 each). Bars represent the average values.
(TIF)

S2 Fig Estimation of *T*. *socialis* swimming velocity. Microalgae inhabit low Reynolds number environments. In this situation, the force acting on the moving cell body is proportional to the product of the viscosity (η), cell size, and moving velocity (v); if we approximate the cell body as a sphere of radius r, then the viscous force acting on it is 6πηrv (Stokes’ formula). Here, we approximate the cross-section of a *C*. *reinhardtii* cell body as a circle with its radius r_c_ (left). We approximate the cross-section of the cell bodies of a *T*. *socialis* colony as a circle with its radius r_t_ inscribed with four *C*. *reinhardtii*, and the colony is a sphere of radius r_t_, r_t_ = r_c_/(√2–1) (right). Suppose the *C*. *reinhardtii* cell swims at v_c,_ and each cell in *T*. *socialis* generates the same force as a *C*. *reinhardtii* cell. In that case, the swimming velocity of *T*. *socialis* can be estimated using the equation v_t_ = 4(6πηr_c_v_c_)/6πηr_c_ ≈ 1.7v_c_. When v_c_ is 150 μm/sec, v_t_ is ~255 μm/sec.
(TIF)

S3 Fig Cilia waveforms of *C*. *reinhardtii* and *T*. *socialis*. The ciliary beating waveforms of live *C*. *reinhardtii* and *T*. *socialis* or their demembranated models reactivated with 1 mM ATP with 10^−3^ Ca^2+^. Ciliary beatings were recorded by a high-speed camera, and ten waveforms per one beat were traced.
(TIF)

S4 Fig Schematic drawing of photoreceptor current measurement. The case of *C*. *reinhardtii* is shown. Cell suspension is put in a cuvette equipped with one electrode on each side. Flash light (λ = 500 nm) is illuminated from one side of the electrode. When the eyespot faces the light source, great amount of Ca^2+^ influxes to the cell (thick arrow), whereas when the eyespot faces opposite to the light source, small amount of Ca^2+^ influxes to the cell (thin arrow). The difference between the Ca^2+^ influx into all cells in the direction of the light source and in the opposite direction is detected as photoreceptor current. Modified from (66).
(TIF)

S5 Fig Channelrhodopsin-1 amino-acid sequence alignment. Amino-acid sequences of *C*. *reinhardtii* ChR1 (XP_001699021.1), *T*. *socialis* ChR-1-like protein (PNH09255.1), *Gonium pectrale* ChR-1 like protein (KXZ47650.1), and *Volvox carteri* ChR1 (ABZ90900.1) were aligned and compared using Clustal Omega. Seven transmembrane domains were colored green (TM1–TM7), and five essential residues for binding retinal were colored blue. The red letter (Phe 265) shows the amino-acid substitution in the retinal binding residues in *T*. *socialis*. Asterisks (*) represent fully conserved, colons (:) represent strongly similar, and periods (.) represent weakly similar residues, respectively.
(TIF)

S6 Fig Swimming trajectories of *T*. *socialis* ISA2-2 at the light-dark border. (A) Schematic showing the swimming trajectory angle analyses. Swimming trajectories were measured for 6 s. We measured the line and the angles (φ) formed by the line connecting position t = 0 to position t = 3. For the colonies swimming across the light-dark border, position t = 3 was set on the borderline. (B) The angle (φ) values in the colonies swimming within the light (L→L) or dark (D→D) areas and those swimming across the borders (L→D or D→L). There were no significant differences between L→L and L→D or D→D and D→L (*p* > 0.1, Student’s *t*-test).
(TIF)

S7 Fig LHCSR1 and LHCSR3 amino-acid sequence alignments. Amino-acid sequences of (A) *C*. *reinhardtii* LHCSR1 (XP_001696125.1) and *T*. *socialis* LHCSR1-like protein (PNH03467.1) or (B) *C*. *reinhardtii* LHCSR3 (XP_001696064) and *T*. *socialis* LHCSR3-like protein (PNH03463.1) were aligned and compared using Clustal Omega. Solid underlines show the peptide used as an antigen and dotted underlines represent corresponding peptide in *T*. *socialis* proteins. Asterisks (*) represent fully conserved, colons (:) represent strongly similar, and periods (.) represent weakly similar residues, respectively.
(TIF)

S1 Movie Swimming *Tetrabaena socialis* NIES-571 colonies. Green light (λ = 525 nm) was illuminated from the left side at ~5 s when the white arrow appears.
(MP4)

S2 Movie Swimming *Chlamydomonas reinhardtii* cells. Green light (λ = 525 nm) was illuminated from the left side at ~5 s when the white arrow appears. Most cells exhibit positive phototaxis.
(MP4)

S3 Movie High-speed movie (400 fps) of the photoshock response of a *Chlamydomonas reinhardtii* cell. After a flash illumination at counter 50, the cell showed backward swimming, and then recovered forward swimming. Replayed at × 1/20 speed.
(MP4)

S4 Movie High-speed movie (400 fps) of a swimming *Tetrabaena socialis* NIES-571 colony. Even after a flash illumination at counter 50, the colony did not change its swimming manner. Replayed at × 1/20 speed.
(MP4)

S5 Movie Spontaneous stop movement of a *Tetrabaena socialis* NIES-571 colony. The colony stopped forward swimming by converting its ciliary waveform from asymmetrical to symmetrical during 2~5 sec and then recovered forward swimming.
(MP4)
