# Correction: The four-celled Volvocales green alga *Tetrabaena socialis* exhibits weak photobehavior and high-photoprotection ability

**DOI:** 10.1371/journal.pone.0355515

**Published:** 2026-08-10

**Authors:** Asuka Tanno, Ryutaro Tokutsu, Yoko Arakaki, Noriko Ueki, Jun Minagawa, Kenjiro Yoshimura, Toru Hisabori, Hisayoshi Nozaki, Ken-ichi Wakabayashi

After this article [1] was published, the corresponding author notified PLOS of errors in Fig 5, S1 Fig, and S3 Fig. Specifically:

- In Fig 5E, the 0, 15, and 30 min control panels are duplicates of the 0, 15, and 30 min +DCMU panels respectively.
- The legend for S1 Fig shows an incorrect sample number for the swimming velocity.
- The *C. reinhardtii* Cell Model +Ca^2+^ traces presented in S3 Fig were created from data published in Fig 1B of a previous article by the same group [2].

The corresponding author stated that the 0, 15, and 30 min time point panels for the control conditions in Fig 5E are incorrect and provided an updated Fig 5 with the correct 0, 15, and 30 min control panels from the original experiments. The original images underlying all panels in Fig 5 are provided in [3].

They further stated that the *C. reinhardtii* Cell Model +Ca^2+^ traces presented in S3 Fig had been inadvertently included in this article and are mirror images of the wild-type data in Fig 1B of [2]. They stated that the *C. reinhardtii* waveform traces were provided as a comparative example to illustrate the Ca^2+^-dependent waveform conversion observed in Tetrabaena and were not used for quantitative analyses. A corrected legend for S3 Fig has been provided to correctly acknowledge [2] as the source of this data. A corrected legend for S1 Fig has been provided to correct the sample sizes to n = 20 for beat frequency and n = 15 for swimming velocity.

The raw data underlying all figures are missing from the list of Supporting Information. The authors have provided this data in [3]. With this correction, all relevant data are now provided.

The Data Availability statement in [1] is incorrect. The correct Data Availability statement is: All relevant data are available from https://zenodo.org/records/21438015.

The *C. reinhardtii* Cell Model +Ca^2+^ traces presented in S3 Fig report material from [2], published in 1997 by Wiley-Liss, which are not offered under a CC BY license and are therefore excluded from this article’s [1] license.

**Fig 5:**
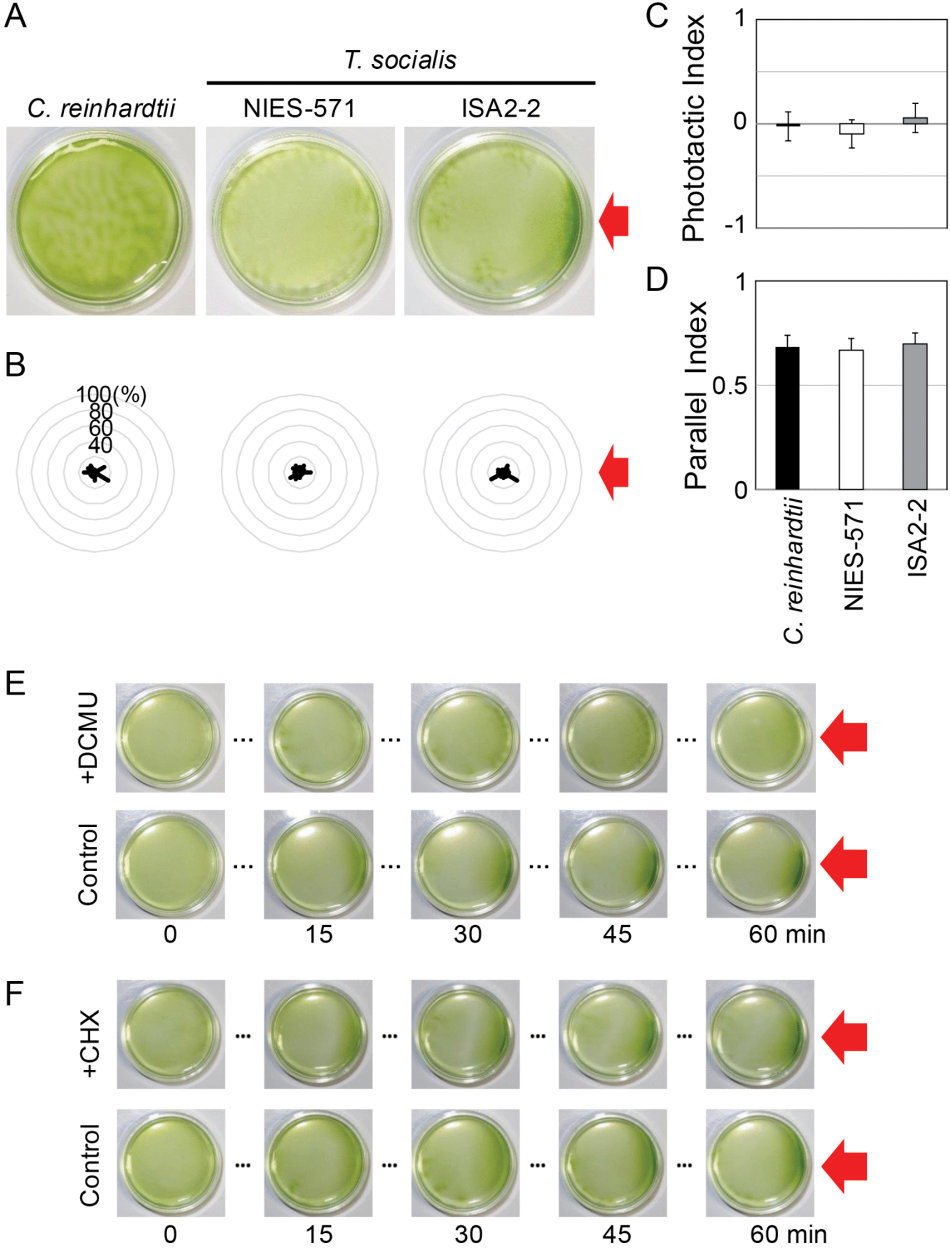
*Tetrabaena socialis* ISA-2-2 showed slow photoaccumulation toward red light in a photosynthesis-dependent manner. (A) Suspensions of wild-type *C*. *reinhardtii* and *T*. *socialis* NIES-571 and ISA2−2 strains were illuminated with red light (λ = 640 nm, 30 μmol photons m^-2^ s^-1^) from the right side for 15 min. (B) Polar histograms representing the percentage of cells/colonies moving in a particular direction relative to the red light illumination (12 bins of 30°; n = 30 cells/colonies per strain) for 3 s following 30-s illumination. (C) Phototactic indices (average of cosθ) and (D) parallel indices (average of |cosθ|) calculated from (B). Values were not significantly different from random swimming (*p* > 0.1; Student’s *t*-*t*est; see Fig 2C and 2D for details). (E, F) Time-lapse observation of *T*. *socialis* ISA2−2 photoaccumulation with (E) DCMU (or ethanol as a control) or (F) cycloheximide (CHX) (or DMSO as a control) treatments for 15 min in the dark. After the red light illumination, images of the Petri dishes were captured every 15 min.

## Supporting information

S1 Fig Motility parameters of *T*. *socialis.* Ciliary beating frequency (left) and swimming velocity of *T*. *socialis* NIES-571 (n = 20 for beat frequency and n = 15 for swimming velocity). Bars represent the average values.
(TIF)

S3 Fig The ciliary beating waveforms of live *C. reinhardtii* and *T. socialis* and of demembranated *T. socialis* cell models reactivated with 1 mM ATP and 10^−3^ M Ca^2+^ were recorded using a high-speed camera and traced. The +Ca^2+^ waveform traces of the *C. reinhardtii* cell model were prepared from data used in images previously published in Fig 1B of Wakabayashi et al. (1997) [2], in which isolated demembranated cilia were reactivated with 20 μM ATP and 10^−4^ M Ca^2+^. Ten waveforms per beat were traced.
(TIF)
