# Supplementary figures and images for: The four-celled Volvocales green alga Tetrabaena socialis exhibits weak photobehavior and high-photoprotection ability
Source: PLoS One. 2021 Oct 26;16(10):e0259138. doi: 10.1371/journal.pone.0259138 (PMC8547699; doi:10.1371/journal.pone.0259138)

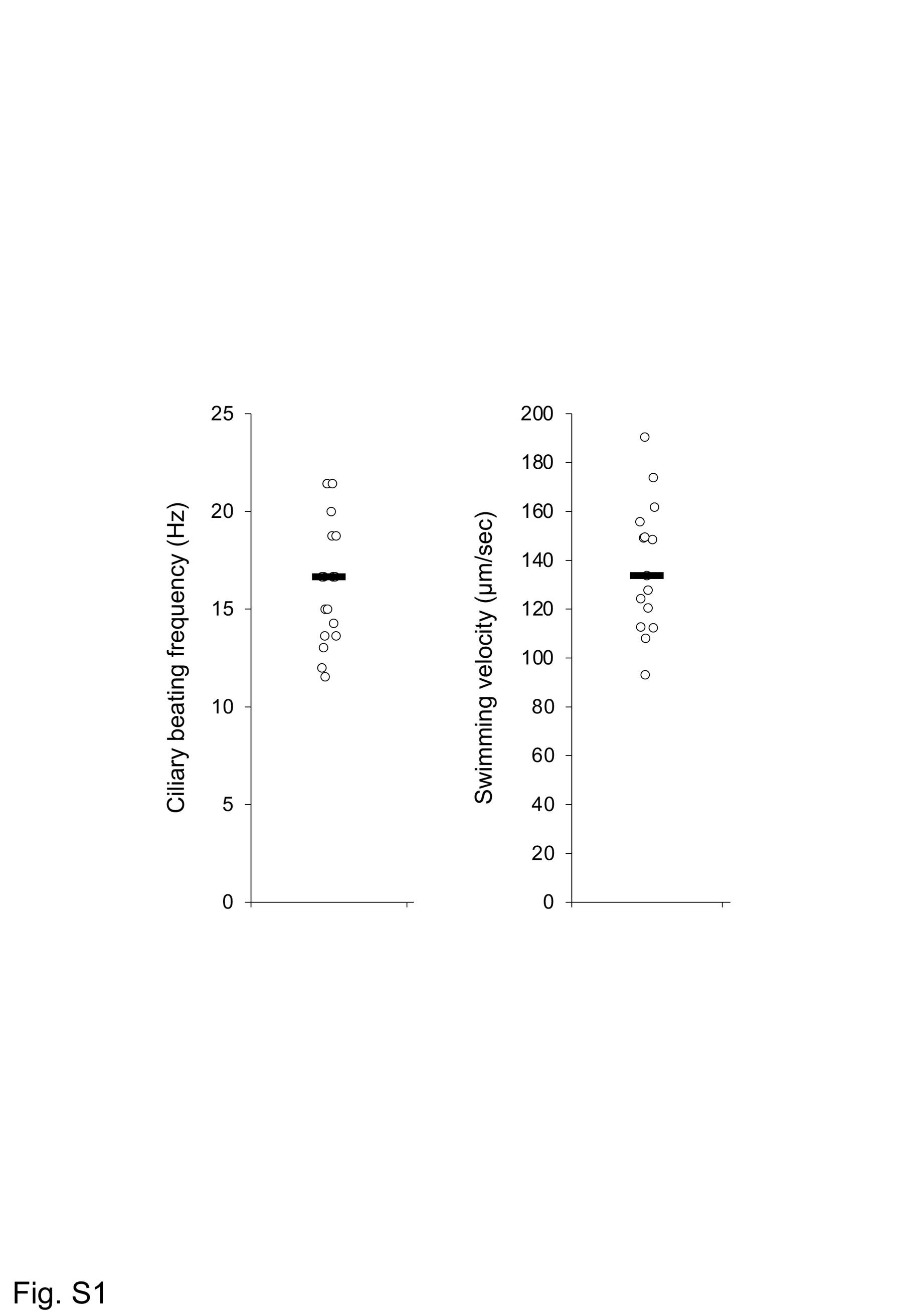

Supplement: S1 Fig — Ciliary beating frequency (left) and swimming velocity of T. socialis NIES-571 (n = 20 each). Bars represent the average values. (TIF) [file pone.0259138.s001.tif]

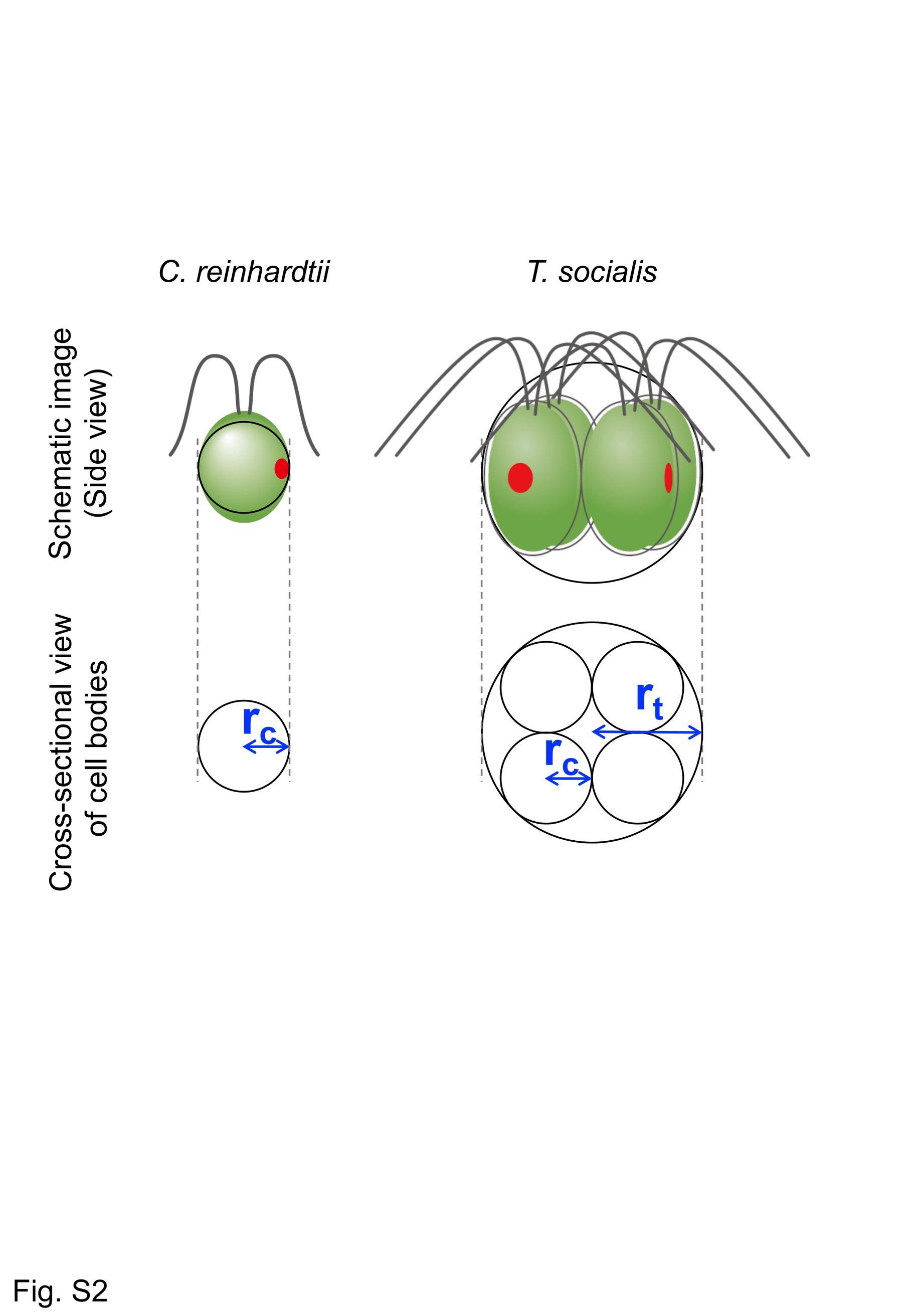

Supplement: S2 Fig — Microalgae inhabit low Reynolds number environments. In this situation, the force acting on the moving cell body is proportional to the product of the viscosity (η), cell size, and moving velocity (v); if we approximate the cell body as a sphere of radius r, then the viscous force acting on it is 6πηrv (Stokes’ formula). Here, we approximate the cross-section of a C. reinhardtii cell body as a circle with its radius rc (left). We approximate the cross-section of the cell bodies of a T. socialis colony as a circle with its radius rt inscribed with four C. reinhardtii, and the colony is a sphere of radius rt, rt = rc/(√2–1) (right). Suppose the C. reinhardtii cell swims at vc, and each cell in T. socialis generates the same force as a C. reinhardtii cell. In that case, the swimming velocity of T. socialis can be estimated using the equation vt = 4(6πηrcvc)/6πηrc ≈ 1.7vc. When vc is 150 μm/sec, vt is ~255 μm/sec. (TIF) [file pone.0259138.s002.tif]

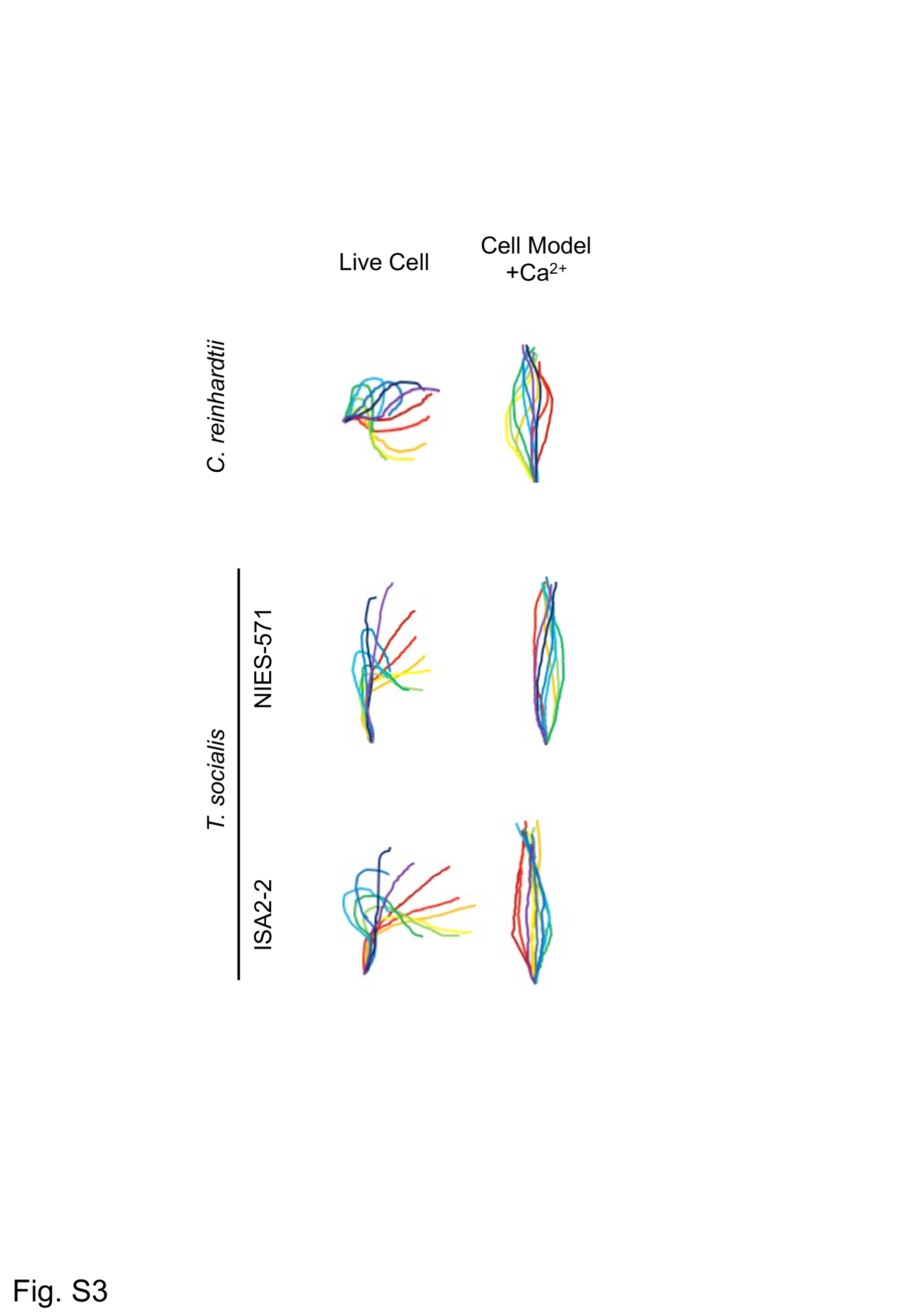

Supplement: S3 Fig — The ciliary beating waveforms of live C. reinhardtii and T. socialis or their demembranated models reactivated with 1 mM ATP with 10−3 Ca2+. Ciliary beatings were recorded by a high-speed camera, and ten waveforms per one beat were traced. (TIF) [file pone.0259138.s003.tif]

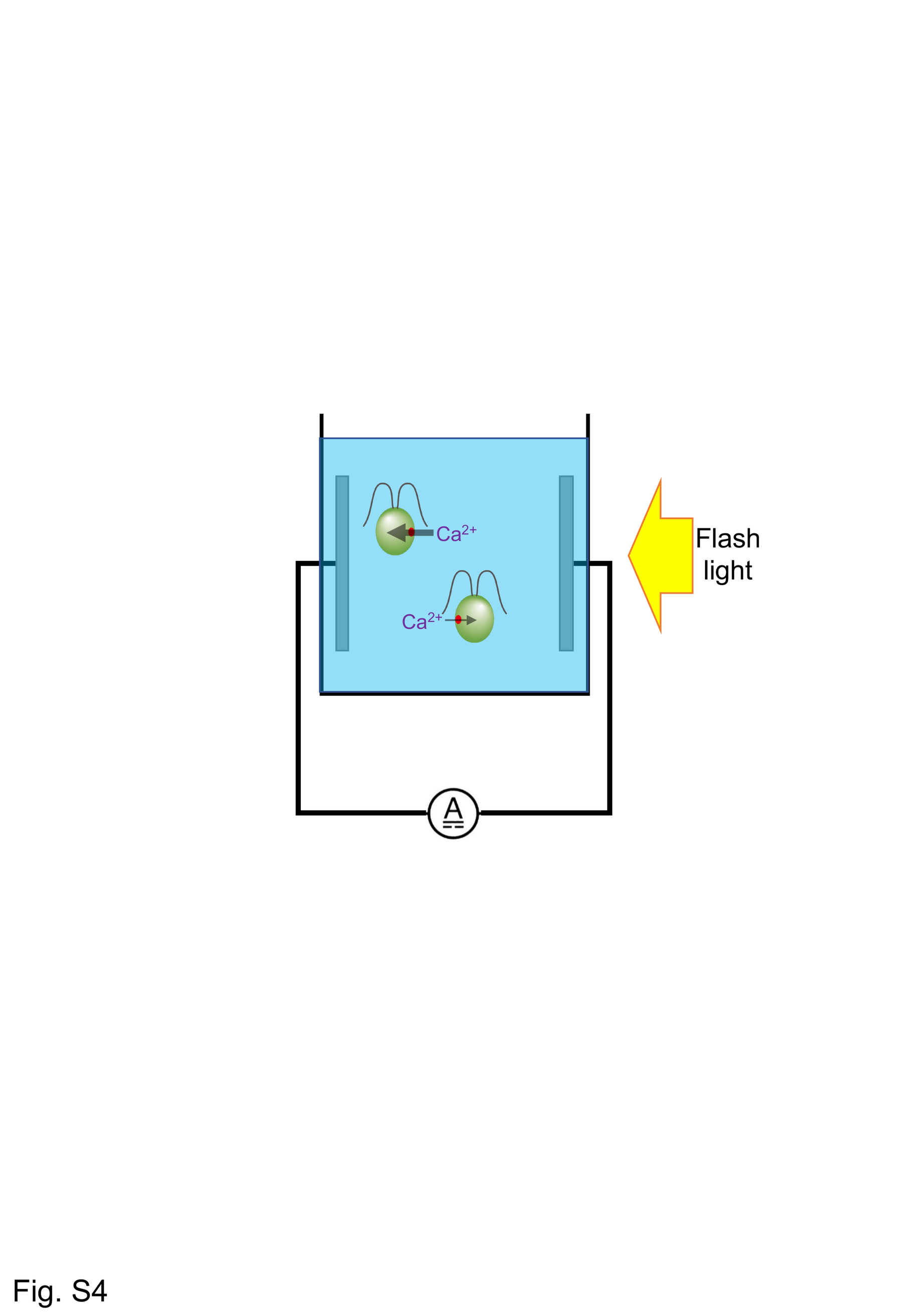

Supplement: S4 Fig — The case of C. reinhardtii is shown. Cell suspension is put in a cuvette equipped with one electrode on each side. Flash light (λ = 500 nm) is illuminated from one side of the electrode. When the eyespot faces the light source, great amount of Ca2+ influxes to the cell (thick arrow), whereas when the eyespot faces opposite to the light source, small amount of Ca2+ influxes to the cell (thin arrow). The difference between the Ca2+ influx into all cells in the direction of the light source and in the opposite direction is detected as photoreceptor current. Modified from (66). (TIF) [file pone.0259138.s004.tif]

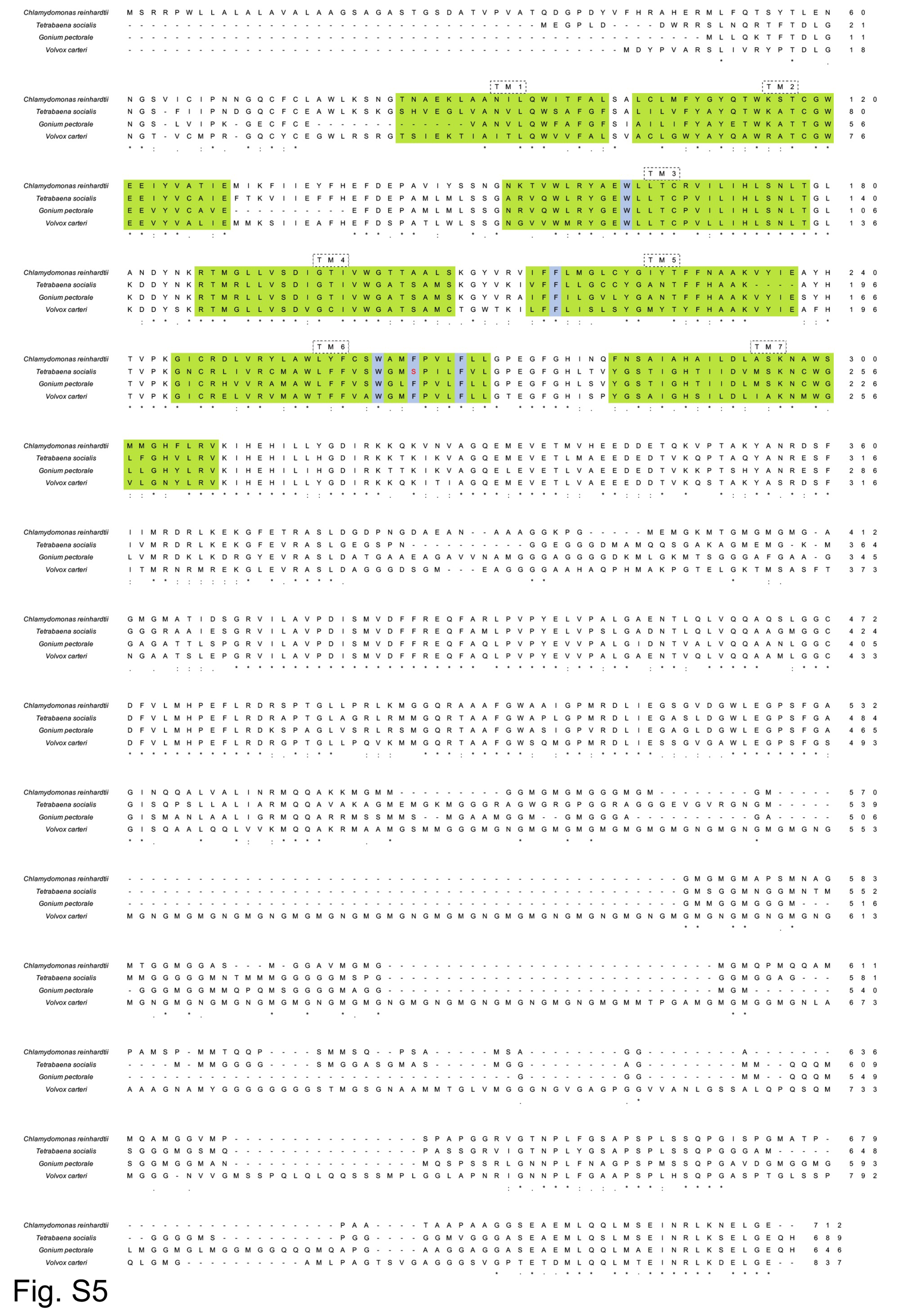

Supplement: S5 Fig — Amino-acid sequences of C. reinhardtii ChR1 (XP_001699021.1), T. socialis ChR-1-like protein (PNH09255.1), Gonium pectrale ChR-1 like protein (KXZ47650.1), and Volvox carteri ChR1 (ABZ90900.1) were aligned and compared using Clustal Omega. Seven transmembrane domains were colored green (TM1–TM7), and five essential residues for binding retinal were colored blue. The red letter (Phe 265) shows the amino-acid substitution in the retinal binding residues in T. socialis. Asterisks (*) represent fully conserved, colons (:) represent strongly similar, and periods (.) represent weakly similar residues, respectively. (TIF) [file pone.0259138.s005.tif]

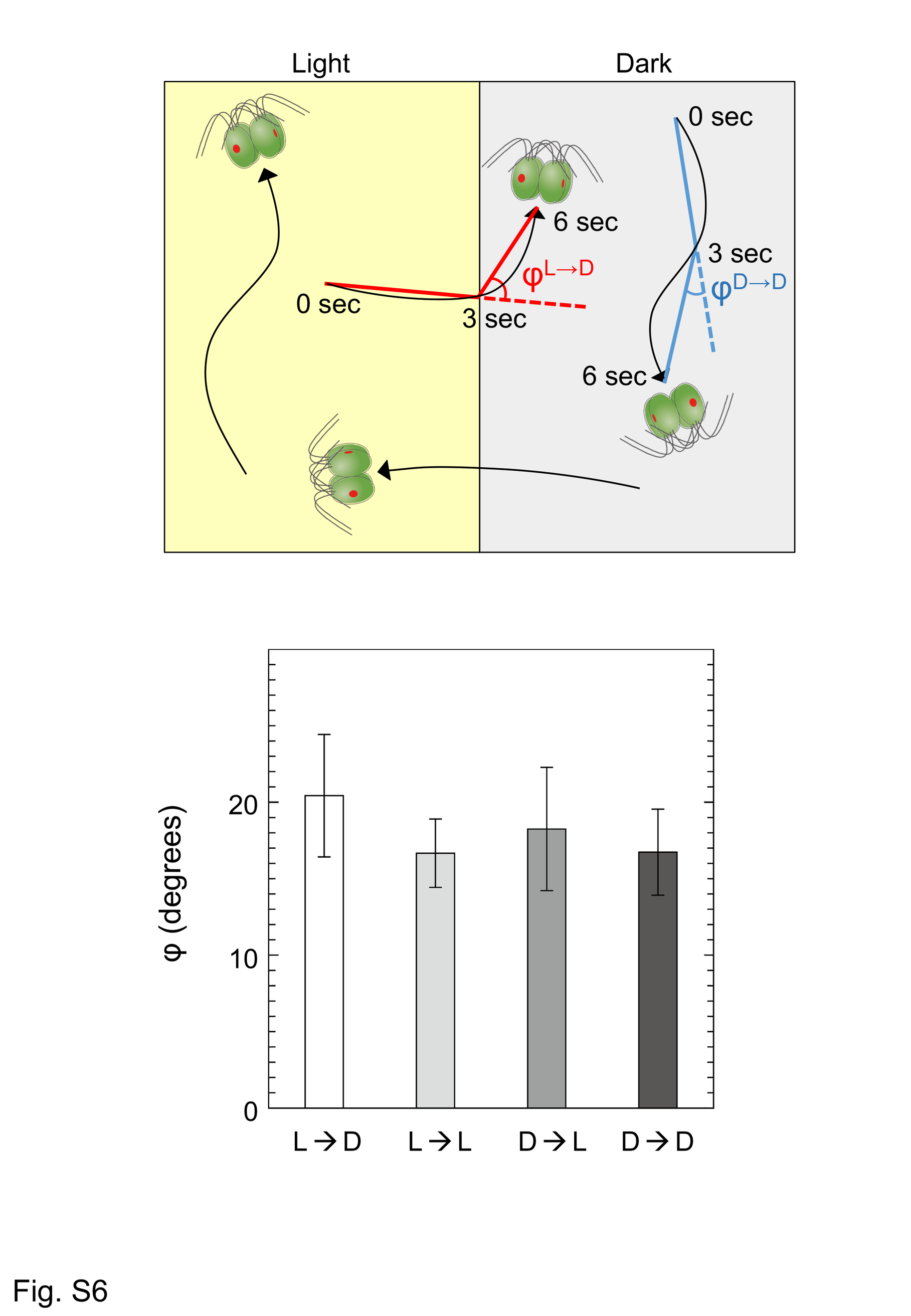

Supplement: S6 Fig — (A) Schematic showing the swimming trajectory angle analyses. Swimming trajectories were measured for 6 s. We measured the line and the angles (φ) formed by the line connecting position t = 0 to position t = 3. For the colonies swimming across the light-dark border, position t = 3 was set on the borderline. (B) The angle (φ) values in the colonies swimming within the light (L→L) or dark (D→D) areas and those swimming across the borders (L→D or D→L). There were no significant differences between L→L and L→D or D→D and D→L (p > 0.1, Student’s t-test). (TIF) [file pone.0259138.s006.tif]

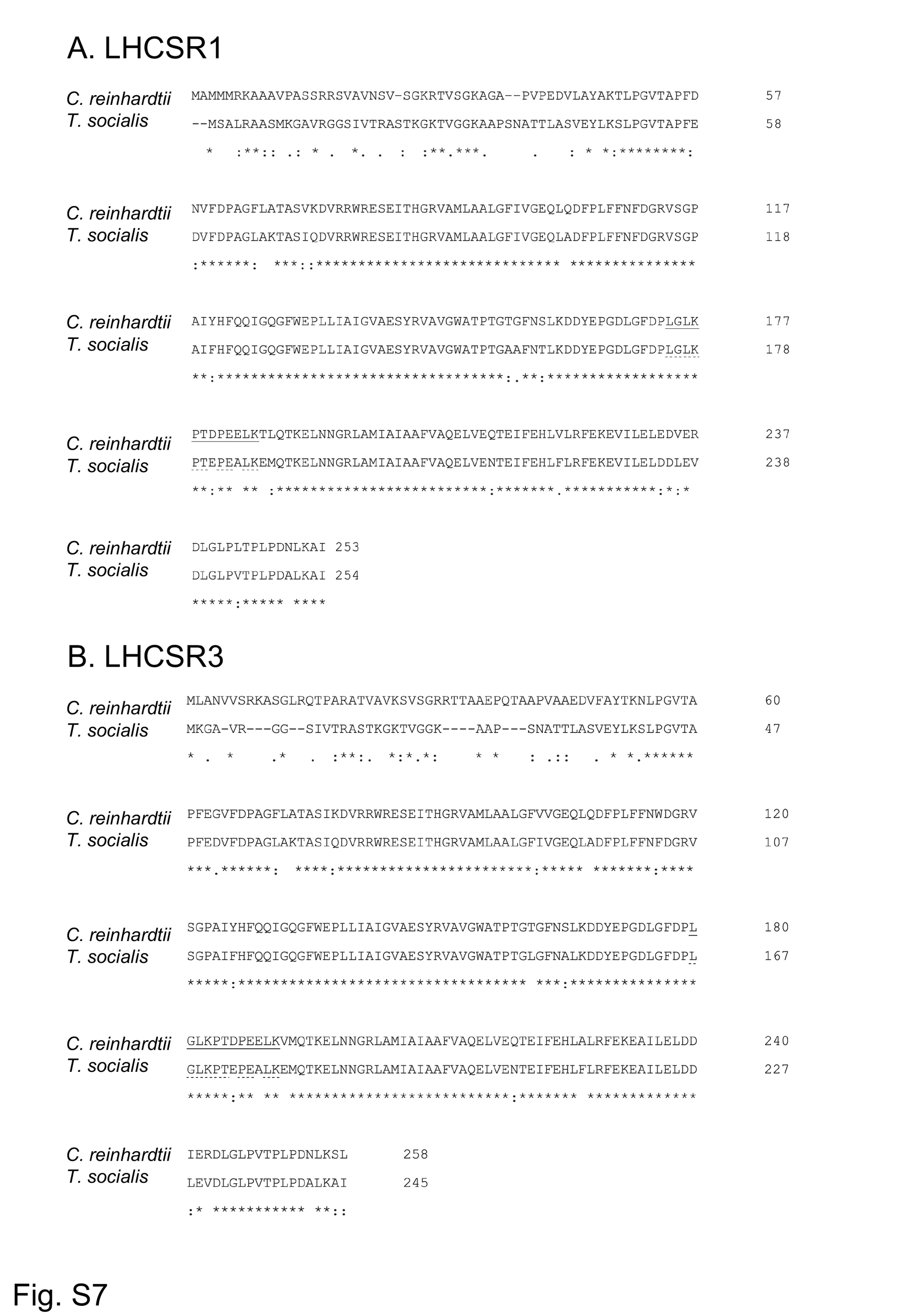

Supplement: S7 Fig — Amino-acid sequences of (A) C. reinhardtii LHCSR1 (XP_001696125.1) and T. socialis LHCSR1-like protein (PNH03467.1) or (B) C. reinhardtii LHCSR3 (XP_001696064) and T. socialis LHCSR3-like protein (PNH03463.1) were aligned and compared using Clustal Omega. Solid underlines show the peptide used as an antigen and dotted underlines represent corresponding peptide in T. socialis proteins. Asterisks (*) represent fully conserved, colons (:) represent strongly similar, and periods (.) represent weakly similar residues, respectively. (TIF) [file pone.0259138.s007.tif]
